# Retraction: Hyponatremia in Acute Stroke: A Comparative Study of Ischemic and Hemorrhagic Stroke Subtypes

**DOI:** 10.7759/cureus.r192

**Published:** 2025-10-03

**Authors:** Zahid Hasan, Murad Ali, Zia Ur-Rehman, Imran Qadar Khattak, Hamza Israr, Aansa Amin, Asim Ali, Amjad Ali

**Affiliations:** 1 Neurology, Mardan Medical Complex, Mardan, PAK; 2 Medicine, Bacha Khan Medical College, Mardan, PAK; 3 Pediatric Cardiology, Maqsood Medical Complex (MMC) General Hospital, Peshawar, PAK; 4 Internal Medicine, Hayatabad Medical Complex Peshawar, Peshawar, PAK; 5 General Internal Medicine, Mardan Medical Complex, Mardan, PAK

This article has been retracted by the Editors-in-Chief due to the presence of extensive methodological flaws, including a lack of formal statistical tests, that seriously undermine the credibility of the study's results and conclusion. The authors disagree with the decision to retract.

